# Lifestyle in adults aged 35 years who were born with open spina bifida: prospective cohort study

**DOI:** 10.1186/1743-8454-1-4

**Published:** 2004-12-10

**Authors:** Gillian M Hunt, Pippa Oakeshott

**Affiliations:** 1 Addenbrooke's Hospital, Cambridge CB2 2QQ, UK; 2 Department of Community Health Sciences, St George's Hospital Medical School, London SW17 ORE, UK

## Abstract

**Background and Methods:**

From 1963 to 1971, 117 babies with open spina bifida were treated non-selectively from birth. In 2002 we reviewed all the survivors by postal questionnaire and telephone call. The aims were to find out how many were living independently in the community or were in open employment or drove a car. In addition to these achievements we recorded health, medication and admissions to hospital and asked how much daily help they needed.

**Results:**

Ascertainment was 100%. There had been 63 deaths, mainly of the most severely affected. The mean age of the 54 survivors was 35 years. The outcome in terms of disability ranged from apparent normality to total dependency. It reflected both the neurological deficit, which had been recorded in infancy in terms of sensory level, and events in the CSF shunt history. Overall about 2 in 5 of the survivors lived independently in the community, 2 in 5 drove a car, 1 in 5 was in competitive employment and 1 in 5 could walk 50 metres.

**Conclusion:**

Although those who survived to age 35 years tended to be less disabled, 2 in 5 continued to need daily care.

## Background

Neurosurgical intervention in babies with open spina bifida had dramatic results in terms of survival. However, the disability and the complications of the survivors were often severe [1-5]. Many efforts were made to enable them to walk, to control their urinary incontinence while safeguarding renal function, and to overcome problems associated with the shunt treatment of hydrocephalus. Promising new methods of management, such as the psoas transplant, urinary diversion and artificial urinary sphincters, which seemed highly successful in the short term, lost favour after 10 or 15 years because of disappointing long-term results. In this unsteady course of progress it is helpful to have a long term follow up of a complete cohort of patients with open spina bifida as a realistic basis for helping parents facing the difficult decisions about termination of an affected pregnancy or treatment after birth.

## Methods

### Patients

In 1963 the Regional Neurosurgical Unit at Addenbrooke's Hospital, Cambridge, England offered treatment to all cases of open spina bifida, without any attempt at selection. Between 1963 and 1971, after a detailed neurological examination, 117 babies (50 male, 67 female) had their open spinal defects closed within 48 hours of birth. A ventriculo-atrial cerebrospinal fluid (CSF) shunt was inserted for hydrocephalus when required.

### Data collection

In 2002 all survivors were surveyed by confidential questionnaire and telephone interview. They were asked about health, disability and achievements in terms of living independently, driving a car and working in open employment. Causes of death for those who had died were obtained from medical records and from the Office of National Statistics. The study was approved by the Cambridge Local Research Ethics Committee.

### Statistical analysis

When first surveyed at the mean age 4 years, the cohort had been classified into four groups according to sensory level to pin prick recorded in infancy [4]. Those with intact sensation right down to the knee (sensory level below L3) had a better short-term outcome than those with no sensation below the umbilicus (sensory level above T11). Mortality and measures of disability and achievement were compared in those with different sensory levels and CSF shunt histories using χ^2^.

## Results

Ascertainment was 100%. Twenty (37%) of the 54 survivors responded to the questionnaire and all survivors or a carer or relative were interviewed by telephone.

### Mortality

**Figure 1:**
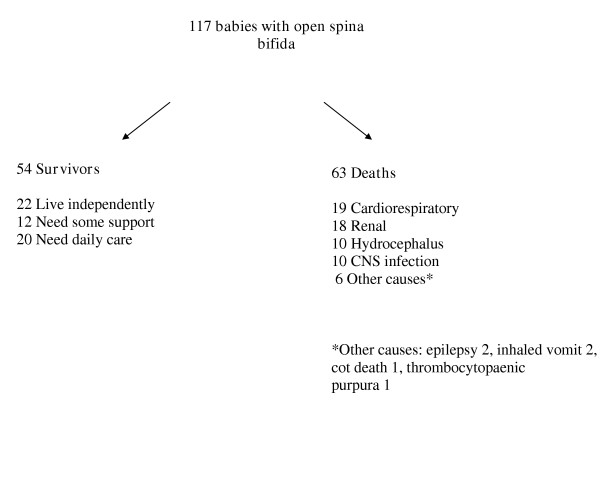
Outcome in open spina bifida at the mean age of 35 years

Figure 1 summarises the outcome for the complete cohort. Sixty-three cases had died, 25 before their first birthday and a further 15 before their fifth. Thereafter the death rate remained constant with an average of 1% of the remainder dying each year [6].

### Survivors

Of the original 117 babies there were 54 survivors (46%) age range 31–38 years. Of these 24 were male and 29 female and there was one who had undergone a gender change from male to female. The disability of the survivors ranged from blindness with paraplegia and double incontinence to apparent normality. Table 1 shows that sensory level in infancy was a predictor of overall disability, the need for a CSF shunt, IQ, and need for a wheelchair or daily care at age 35 years.

**Table 1:** Sensory level in infancy related to disability at the mean age of 35 years in 54 survivors with spina bifida

**Sensory level n = 54 (%)**	**Below L_3 _n = 24**	**L_3_-T_11 _n = 15**	**Above T_11 _n = 12**	**Asymmetrical n = 3**	**χ^2 ^for trend^(1)^**
Severe disability^(2) ^n = 20 (37)	2	6	12	0	p < 0.0001
CSF shunt n = 46 (85)	17	15	11	3	p < 0.05
IQ < 80 n = 15 (28)	3	4	6	2	p = 0.05
Wheelchair n = 38 (70)	9	14	12	3	p < 0.0001
Daily care needed n = 20 (37)	5	6	9	0	p < 0.05
Lack of achievement^(3) ^n = 27 (50)	7	9	10	2	p < 0.01

Notes: ^(1) ^Patients with lower sensory levels have less disability. Asymmetrical sensory level excluded from the analysis.

^(2) ^Severe disability defined as very poor mobility and incontinent with additional handicaps including low IQ, epilepsy, visual defects (2 blind), severe spinal deformity and pressure sores.

^(3) ^Lack of achievement in terms of living independently, driving a car or working in open employment.

### Mobility

Only 16 (30%) remained community walkers defined as being able to walk ≥50 metres with or without aids. Ten of the 16 could walk at least a kilometre. Table 2 shows the deterioration in walking since childhood and its relationship to sensory level. By the age of 35 there was only one community walker with a sensory level as high as L3. But of those with a low sensory level of L5 and below, 88% (14/16) remained walkers. In terms of motor function, 30 had been recorded as having bilateral quadriceps activity in infancy, but only 53% of them (16/30) remained walkers at the age of 35 years.

**Table 2:** Influence of sensory level and age on walking in 54 survivors with spina bifida

Sensory level in infancy	n = 54	Walkers^1 ^at age 9 n = 31 (57%)	Walkers at age 35 n = 16 (30%)
Above T11	n = 12	0	0
T11-L3	n = 15	5	1
L4	n = 8	8	1
L5-S2	n = 6	6	5
No sensory loss	n = 10	10	9
Asymmetrical loss	n = 3	2	0

^1 ^Walkers defined as able to walk ≥50 metres using aids if required.

Survivors with lower sensory levels more likely to be walkers. (χ^2 ^for trend p < 0.0001 for both age 9 and age 35. Asymmetrical sensory loss excluded from the analysis.)

### Cerebrospinal fluid (CSF) shunts

Of the 54 survivors, eight (15%) never had a shunt, seven of whom had little or no disability and a sensory level below L3. The remaining 46 had had a ventriculo-atrial shunt inserted. In 16 the shunt had never been revised. The other 30 had had a total of 104 revisions: for shunt insufficiency (65), infection (15), detachment (14), extrusion or leaking wound of back (5), unknown (5). In 9 patients revisions were done only before the age of 2 (mean 1.3 revisions, range 1–3), and in 21 between the ages of 2 and 35 (mean 3.1 revisions, range 1–14). Elective revisions were not performed; and shunts were inserted or revised only in response to definite clinical need. Of those who had revisions, 75% had had symptoms of raised intracranial pressure.

### Health

Nearly half of the survivors had been in hospital during the previous 5 years. The main reasons were urological (7 patients), neurosurgical (3), and sepsis (7). Pressure sores were responsible for four of the admissions for sepsis, and 12 patients were currently being treated at home for pressure sores. Only 11 patients (20%) were fully continent of bladder and bowel without the use of catheters or appliances. Two patients needed nocturnal respiratory support, two were totally blind following shunt dysfunction and four others had severe visual defects. Endocrine conditions were common: two patients had diabetes mellitus, one had adrenal hyperplasia, one had primary azoospermia and six had had precocious puberty. Twenty-four patients were on long-term therapy: antihypertensives (12), anticonvulsants (10), antibacterials (10) and antidepressants (4). Eight patients needed regular analgesics for musculo-skeletal pain, mainly backache.

### Parenthood

Seven women and two men had become parents. One man had minimal disability and no detectable sensory loss; the other had undergone percutaneous epididymal sperm aspiration followed by intracytoplasmic sperm injection. None of the 13 children had visible spina bifida.

### Residence and dependency

Twenty-two individuals (41%) lived independently in the community, 11 of them used wheelchairs. A further 12 (22%) were personally independent but had supervision and help when required. The remaining 20 (37%) needed help daily for dressing, shaving, toilet or nursing care (mainly pressure sores). Ten of these still lived with a parent now aged 52–77, two women were in the care of their partners, five were in residential establishments and three lived in the community with help from social services (Table 3).

**Table 3:** Where are the 54 survivors living?

Residence and Dependency	Number of individuals	Percentage
Independent living	22	41
Sheltered environment with help available	12	22
Dependent on daily help	20	27

### Car drivers

Twenty-nine (54%) of the survivors had passed the driving test, but 9 had discontinued driving. Eleven others were unfit to drive on account of poor sight (3), epilepsy (3) or severe cognitive or perceptual defects (5).

### Employment

Nine men and four women were in open employment. All had an IQ ≥80 and five used wheelchairs. Three did clerical work, three were teachers, two were unskilled manual workers and the remainder were a business executive, accountant, engineer, van driver and builder. Three were studying in addition to working full time. Three men and two women were in sheltered employment.

### Lifestyle and achievements

Twenty-seven survivors (50%) had one or more achievements in terms of living completely independently in the community (22), driving a car (20) or working in open employment (13). Achievements were related to sensory level in infancy and to shunt history (Tables 1 and 4). All but one of the 8 patients without a shunt and 75% (12/16) of those in whom the shunt was never revised were classified as achievers. They lived independently or drove a car or worked in open employment compared with 40% (4/10) of those needing revision at age <2 and 20% (4/20) of those revised after age 2 (p < 0.01). Table 5 shows that late revisions of shunt after the age of 2 were also associated with a birth head circumference ≥90th centile relative to birthweight, a history of symptoms of raised intracranial pressure, visual defects and the need for daily care.

**Table 4:** Lifestyle related to history of CSF shunt in 54 survivors at the mean age of 35 years

**Lifestyle**	**No shunt n = 8**	**Shunt not revised^1 ^n = 16**	**Shunt revised age <2 n = 10**	**Shunt revised age 2–35 n = 20**
Living independently n = 22 (41%)	7	9	4	2***
Driving a car n = 20 (37%)	5	8	4	3*
In open employment n = 13 (24%)	3	6	3	1*
Any achievement^(2) ^n = 27 (50%)	7	12	4	4**

χ^2 ^comparing those with shunt revisions aged 2–35 with those never revised or revised at age <2. *p < 0.05 **p < 0.01, ***p < 0.001. Those with no shunts excluded from the analysis.

^(1) ^Shunts were only inserted or revised in response to definite clinical need such as symptoms or signs of raised intracranial pressure.

^(2)^Any achievement: in terms of living independently, driving a car or working in open employment.

**Table 5:** Features related to CSF shunt history in 54 patients with open spina bifida at the mean age of 35 years

**Features**	**No shunt n = 8**	**Shunt not revised n = 16**	**Shunt revised age <2 n = 10**	**Shunt revised age ≥2 n = 20**	**χ^2 ^for trend**
Birth head circumference ≥90 centile n = 10	0	1	1	8	p = 0.05
History of symptoms of raised intracranial pressure n = 23	0	1	5	17	p < 0.0001
Visual defects (mainly squint) n = 33	3	7	6	17	p < 0.05
Daily care needed n = 20	1	2	2	15	p < 0.001

## Discussion

By the mean age of 35 years, over half the cohort had died, mainly the most disabled. About 40% of the survivors lived independently, 20% needed some support and 40% needed daily care. Lifestyle and achievements depended on the degree of disability, which could have been forecast from sensory level recorded in infancy, indicating the extent and severity of the neural deficit. Data from this cohort show that babies with sensation below the knee (L3) are unlikely to be seriously disabled and could be achievers in adulthood. Babies who cry during a routine heel prick have a sensory level of S1 or below and are likely to remain community walkers in adulthood. Those with sensation to pin prick in the saddle area (S2, 3, 4) are likely to have bladder and bowel control.

Events in the history of the CSF shunt also had a profound effect on outcome and achievement. Revisions of shunt were associated with poor achievement particularly when the revisions were needed after the age of 2. The cranial sutures have usually fused by the age of 2 after which the skull is less expansile rendering the brain more susceptible to pressure [7]. Table 5 shows that most of those who had revisions after the age of 2 had had symptoms or signs of raised intracranial pressure. By contrast an uneventful shunt history was sometimes associated with remarkable achievement despite severe disability. This may imply that it is the raised intracranial pressure which has the adverse long term effect on achievement and enterprise [8].

The main strength of the study is the community basis, which provides social as well as clinical data enabling the realities of adulthood to be seen against the optimistic forecasts of the early years [9]. Less than half of the survivors were still attending hospital. Thus a hospital-based study would have given an incomplete picture. As the patients grew older, the reduction in support, rehabilitation and encouragement from dedicated physiotherapists, parents and other carers revealed an outcome which was related to the patient's own motivation and enterprise as well as to the basic neurological deficit [8]. The main limitation of the study is that the very long follow up relates to some treatments, which have been superseded. Improvements in the diagnosis and management of renal and neurological problems have halved the mortality by the age of 5 [10,11], but have less influence on long term disability.

Although outcome in childhood of early operated spina bifida has been widely reported [1-5,11], there are few studies of long term outcome. McLone has argued strongly that prognosis is improving due to advances in treatment [12]. Our results may not predict outcome using today's standards of care. However, a recent survey from McLone's group of a cohort of 118 adults aged 20–25 with 16% loss to follow up, found continuing deterioration and a formidable number of neurosurgical and spinal operations [13]. Ours is the only 35-year prospective study of open spina bifida with 100% ascertainment by the same independent observer.

## Conclusions

These data may help health professionals counselling parents of a child with spina bifida. They show a range of possible outcomes in adulthood when parents may no longer be able or willing to look after their child [14-16]. Two out of 5 survivors continue to need daily care. Advances in treatment may have improved prognosis, but the most important predictor remains the basic neurological deficit. Those looking after patients with spina bifida need to know both their long term potential and the limitations of treatment in order to focus on realistic goals [17].

## Competing interests

The authors declare that they have no competing interests.
